# Thermodynamic Origin of Differential Excipient-Lysozyme Interactions

**DOI:** 10.3389/fmolb.2021.689400

**Published:** 2021-06-11

**Authors:** Jas Kalayan, Robin A. Curtis, Jim Warwicker, Richard H. Henchman

**Affiliations:** ^1^ Manchester Institute of Biotechnology, The University of Manchester, Manchester, United Kingdom; ^2^ Department of Chemistry, The University of Manchester, Manchester, United Kingdom; ^3^ Departments of Chemical Engineering and Analytical Science, The University of Manchester, Manchester, United Kingdom; ^4^ Division of Molecular and Cellular Function, School of Biological Sciences, Faculty of Biology, Medicine and Health, The University of Manchester, Manchester, United Kingdom; ^5^ Faculty of Medicine and Health, Sydney Medical School, The University of Sydney, Sydney, NSW, Australia

**Keywords:** statistical mechanics, entropy, free energy methods, multiscale, metadynamics method, protein-protein binding, protein-excipient binding, protein hydration

## Abstract

Understanding the intricate interplay of interactions between proteins, excipients, ions and water is important to achieve the effective purification and stable formulation of protein therapeutics. The free energy of lysozyme interacting with two kinds of polyanionic excipients, citrate and tripolyphosphate, together with sodium chloride and TRIS-buffer, are analysed in multiple-walker metadynamics simulations to understand why tripolyphosphate causes lysozyme to precipitate but citrate does not. The resulting multiscale decomposition of energy and entropy components for water, sodium chloride, excipients and lysozyme reveals that lysozyme is more stabilised by the interaction of tripolyphosphate with basic residues. This is accompanied by more sodium ions being released into solution from tripolyphosphate than for citrate, whilst the latter instead has more water molecules released into solution. Even though lysozyme aggregation is not directly probed in this study, these different mechanisms are suspected to drive the cross-linking between lysozyme molecules with vacant basic residues, ultimately leading to precipitation.

## 1 Introduction

Protein therapeutics are increasingly being developed in the biopharmaceutical industry to combat a wide range of diseases (Dimitrov, 2012; Faber and Whitehead, 2019). Compared with traditional small drug molecules, the large and complex structures and marginal stability of biomolecules make necessary the development of sophisticated formulations which can stabilise the structure of such therapeutics to ensure their safe administration and efficacy (Wang, 2015). Without the correct formulation conditions, proteins are prone to aggregation, precipitation or phase separation (Moussa et al., 2016). The modulation of protein-protein interactions (PPIs) in formulations is commonly achieved by the addition of small molecules, termed excipients. However, a lack of understanding of how excipients operate is hampering further development because such systems comprise multiple, transient, weak interactions between proteins, excipients, other ions and water in solution (Falconer, 2019). This is an extension of the difficulties in understanding aqueous electrolytes, where behaviour even of simple salt solutions is not well explained, particularly at higher concentrations (Collins, 1997). It is therefore important to develop new strategies to identify the effects of excipients on protein stability to improve therapeutic formulations in the biopharmaceutical industry.

**FIGURE 1:**
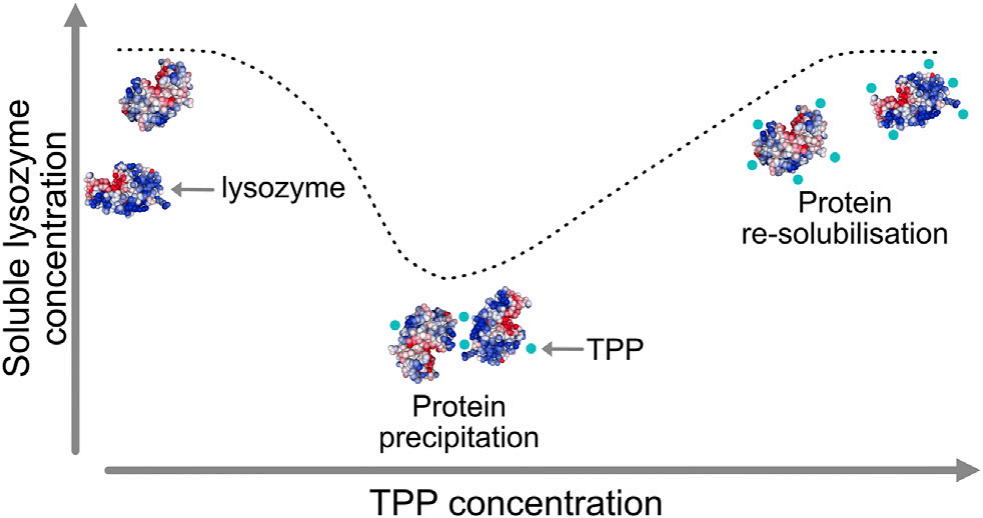
Lysozyme precipitation at intermediate tripolyphosphate (TPP) concentrations and re-solubilisation at higher concentration.

A particularly intriguing phenomenon relating to the mechanism of excipients operation was recently revealed for the case of the protein lysozyme interacting with two excipients, namely tripolyphosphate (TPP) and citrate (CIT). Lysozyme is a small, stable protein that is widely studied and known to remain largely folded in both experiments and simulations and so does not require extensive sampling of protein tertiary structural changes. Experimental data, as illustrated schematically in Figure 1, has shown how TPP excipients cause lysozyme to precipitate out of solution and then resolubilise at higher TPP concentration, but similarly charged CIT excipients do not cause lysozyme precipitation at any concentration (Bye and Curtis, 2019).

The reason for these trends is not clearly understood, but it is hypothesised that TPP cross-links lysozyme due to more polarised, multi-directional P-O^-^ bonds which form stronger interactions with basic residues. Conversely, CIT may have sterically hindered hydrogen bonding with donors on the protein surface, which prevents cross-linking between multiple lysozyme molecules (Bye and Curtis, 2019). In this work we concentrate on the differences between TPP and CIT at the excipient concentration where TPP causes lysozyme precipitation but CIT does not.

Computational methods can contribute much to understanding protein-excipient binding, given the high level of detail that they provide. However, conventional methods for molecular binding are less suitable for such problems because they tend to be aimed at systems in which there are specific binding sites between a substrate and its target, owing to the long-standing influence of structure-based drug design. Rather, it is typically the case for excipients that they bind non-specifically to protein surfaces and in some cases not even directly to proteins at all (Zalar et al., 2020). This diminishes the usefulness of computational techniques such as docking studies and alchemical free-energy methods in favour of ensemble-based methods such as molecular dynamics (MD) or Monte Carlo (MC) simulations that are able to sample the wide range of relevant configurations (Schames et al., 2004; Spyrakis et al., 2015; Yu et al., 2018; Barata et al., 2016). Given the large number of possible configurations that need to be sampled, more efficient enhanced-sampling simulation methods are required. Most enhanced sampling methods for proteins have been conducted to explore protein folding, allostery or protein-ligand binding (Singh et al., 2017; Du et al., 2017; Barducci et al., 2013; Verona et al., 2017). Studies of multiple ligands interacting with a protein surface have shown how increased sampling produces binding affinities comparable to experiment (Troussicot et al., 2015) as well as how co-solutes can affect dissociation of proteins (Banerjee et al., 2019). The use of multiple simulations starting from different poses of two proteins has been shown to be effective in reproducing native protein-protein association structures and in the prediction of new bound configurations (Plattner et al., 2017). Such methods may yield the extent of excipient binding via brute-force probabilities between bound and unbound but are unable to explain the calculated stability for different excipients. Achieving this requires more detailed strategies such as determining how all of the molecules in a system contribute to the total Gibbs free energy. However, most such studies on proteins have focused on the contribution of water molecules or the protein itself (Tarek and Tobias, 2000; Gerogiokas et al., 2016; Chong and Ham, 2017).

Here we seek to understand how lysozyme is differentially stabilised by the excipients TPP and CIT to help explain its aggregation behaviour by applying a free energy method that calculates energy and entropy directly from a simulation called EE-MCC (Energy-Entropy Multiscale Cell Correlation). Energy is calculated directly from the system Hamiltonian by summing over per-atom energies. Entropy is calculated for all molecules in the system from forces and coordinates at multiple length scales using MCC, which has been applied to liquids (Higham et al., 2018; Ali et al., 2019), chemical reactions (Ali et al., 2020), and proteins (Chakravorty et al., 2020). The three length scales employed here from smallest to largest are 1) water and monatomic ions, 2) excipients and residues, and 3) the whole protein, which are classified here as united-atom, monomer, and polymer levels, respectively. We examine the Gibbs free energy for mixing a lysozyme dimer with TRIS buffer and counterions with five excipient molecules, either TPP or CIT, together with counterions, corresponding to the concentration at which TPP-induced aggregation is detected experimentally. Protein-excipient configurations are sampled using metadynamics and multiple-walker simulations to allow the exploration of more transient interactions than would be possible using a conventional molecular dynamics simulation. Although, the large phase space required to sample solute interactions remains a bottle-neck, we observe several important differences between CIT and TPP containing solutions that may help explain their effects on lysozyme aggregation.

## 2 Methods

### 2.1 Multiscale Cell Correlation Entropy Theory

In MCC, entropy *S* is calculated in a multiscale fashion in terms of cells of correlated units. The total entropy is calculated as a sum of components $S_{ij}^{kl}$ using the equation

$$S=\overset{\text{molecule}}{\underset{i}{\sum }}\overset{\text{level}}{\underset{j}{\sum }}\overset{\text{type}}{\underset{k}{\sum }}\overset{\text{motion}}{\underset{l}{\sum }}S_{ij}^{kl}$$ (1)

In this equation, *S* is calculated for each kind of molecule *i*, at the appropriate length scales *j* for each molecule, in terms of translational or rotational motion *l* over all units at that level, and in terms of vibration or topography *k* for each type of motion. Vibrational entropy relates to the average size of energy wells for that unit while topographical entropy relates to the probability distribution of the energy wells. Length scales are defined at the united-atom (UA), monomer (M) and polymer (P) levels for the protein, at the UA and M levels for excipients, and the UA level for water and monatomic ions.

#### 2.1.1 Vibrational Entropy

The entropy of internal molecular vibrations of a unit in the x,y,z directions at each length scale are calculated from the frequencies $\nu_{i}$ of $N_{\text{vib}}$ number of translational or rotational vibrations using

$$S^{\text{vib}}=k_{\text{B}}\sum_{i=1}^{N_{\text{vib}}}\left(\frac{h\nu_{i}/k_{\text{B}}T}{e^{h\nu_{i}/k_{\text{B}}T}-1}-\text{ln}\left(1-e^{-h\nu_{i}/k_{\text{B}}T}\right)\right)$$ (2)

where *h* is Planck’s constant, $k_{\text{B}}$ is Boltzmann’s constant and *T* is temperature. Frequencies $\nu_{i}$ are obtained from the eigenvalues $\lambda_{i}$ of diagonalised mass-weighted force and inertia-weighted torque covariance matrices using

$$\nu_{i}=\frac{1}{2\pi }\sqrt{\frac{\lambda_{i}}{k_{\text{B}}T}}$$ (3)

The forces and torques are rotated into appropriate reference frames defined by the type of unit as shown in Supplementary Table S1. In the mean-field approximation, forces at the polymer level and torques at all levels are halved. For all but the highest length scale in each molecule, vibrational entropies associated with the six smallest frequencies in the force covariance matrix are removed to avoid double counting translation and rotation at the higher level.

#### 2.1.2 Conformational Entropy

Translational topographical entropy at the united-atom level is otherwise known as conformational entropy. It is calculated from the probabilities $p_{i}$ of sets of conformers for all dihedrals in the monomer using

$$S_{\text{UA}}^{\text{topo}}=-k_{\text{B}}\sum_{i=1}^{N_{\text{conf}}}p_{i}\text{ln}p_{i}$$ (4)

where $N_{\text{conf}}$ is the number of sets of conformers. Each conformer *i* is assigned by discretising the distribution of observed dihedral angles into $30^{\circ }$ bins, conformers are centered on peaks defined at bins for which two adjacent bins are less populated and no peaks are closer than $60^{\circ }$, and each dihedral angle is assigned to the nearest peak.

#### 2.1.3 Orientational Entropy

The entropy arising from water molecule orientations with respect to neighbouring united atoms is a generalisation from before (Higham et al., 2018) that captures anisotropy due to the presence of solutes. Orientational entropy is calculated using

$$S_{\text{UA}}^{\text{orient}}=k_{\text{B}}\sum_{c}p\left(c\right)\text{ln}\left[{\left(N_{\text{eff}}\pi \right)}^{\frac{3}{2}}p\left({\text{HB}}_{\text{av}}\right)/\sigma \right]$$ (5)

where *c* is the coordination-shell type of a water molecule and σ is the symmetry number of water, equal to 2. The average bias in hydrogen bonds (HBs) $p\left({\text{HB}}_{\text{av}}\right)$ is a weighted average over the HB biases to all $N_{c}$ neighbours in the coordination shell

$$p\left({\text{HB}}_{\text{av}}\right)=\frac{\sum_{n}p\left({\text{HB}}_{i}\right)N_{i}}{N_{c}}$$ (6)

where $N_{i}$ is the number of neighbours of type *i* in the coordination shell and $p\left({\text{HB}}_{i}\right)$ is the bias in hydrogen-bonding with neighbour *i* given by

$$p\left({\text{HB}}_{i}\right)=\frac{p\left({\text{D}}_{i}\right)}{p\left({\text{D}}_{i}\right)+p\left({\text{A}}_{i}\right)}\times \frac{p\left({\text{A}}_{i}\right)}{p\left({\text{D}}_{i}\right)+p\left({\text{A}}_{i}\right)}$$ (7)

where $p\left({\text{A}}_{i}\right)$ is the probability of accepting from neighboring species *i* over all other neighbours being accepted from and $p\left({\text{D}}_{i}\right)$ is the probability of donating to neighbouring species *i* over all other neighbours being donated to. For bulk water, $p\left({\text{D}}_{i}\right)=p\left({\text{A}}_{i}\right)=1$, but when solutes are nearby, the HBs may be biased in one direction, thus reducing the number of orientations of the water molecule. The effective number of neighbours $N_{\text{eff}}$ that a water molecule can accept and donate HBs with is calculated from the HB probabilities relative to the unbiased value 0.25 as

$$N_{\text{eff}}=\underset{i}{\sum }\frac{p\left({\text{HB}}_{i}\right)N_{i}}{0.25}$$ (8)

Neighbouring water molecules are identified by the solute they are closest to when in the solute coordination shell and labeled as bulk otherwise. Neighbouring solutes are identified as their particular united atoms that are proximal to the water molecule. Contacts between united atoms are defined using the relative angular distance (RAD) algorithm (Higham and Henchman, 2016). HBs are defined topologically (Henchman and Irudayam, 2010; Irudayam et al., 2010; Irudayam and Henchman, 2011) based on the acceptor with the most negative $q\text{D}q\text{A}/r^{2}$ for each donor, where qD and qA are the charges of the donor and acceptor, respectively, and *r* is the distance between them. We use a qualitative proxy for positional entropy of the mixing molecules by considering the number of solute-water contacts. We neglect the contribution of the orientational entropy of the excipients, assuming for these weakly binding molecules that it does not change significantly.

### 2.2 Molecular Energy

The potential (*U*) and kinetic (*K*) energies per atom *i* from simulation are summed to give the enthalpy *H* of a given unit

$$H\approx \sum_{i}\left(K_{i}+U_{i}\right)$$ (9)

ignoring the negligible pressure-volume term.

### 2.3 Water Molecule Assessment Based on Coordination Shell Neighbours

The immediate environment of a water molecule is defined by what molecules are in its first coordination shell. This is used to assess the free energy of water molecules depending on what solutes they interact with. As there are many possible local configurations of a coordination shell, we simplify the definition of local water environments by defining five categories of water contacts with various combinations of excipients and counterion or lysozyme molecules as follows:

1) ${\text{W}}_{\text{P}}$: UAs from only one protein.
2) ${\text{W}}_{\text{PP}}$: UAs from two proteins.
3) ${\text{W}}_{\text{EP}}$: UAs of one protein and any excipient or counterion.
4) ${\text{W}}_{\text{EPP}}$: UAs of two proteins and any excipient or counterion.
5) ${\text{W}}_{\text{E}}$: UAs of any excipient or counterion.

### 2.4 Change in Free Energy for Excipient Binding

**FIGURE 2:**
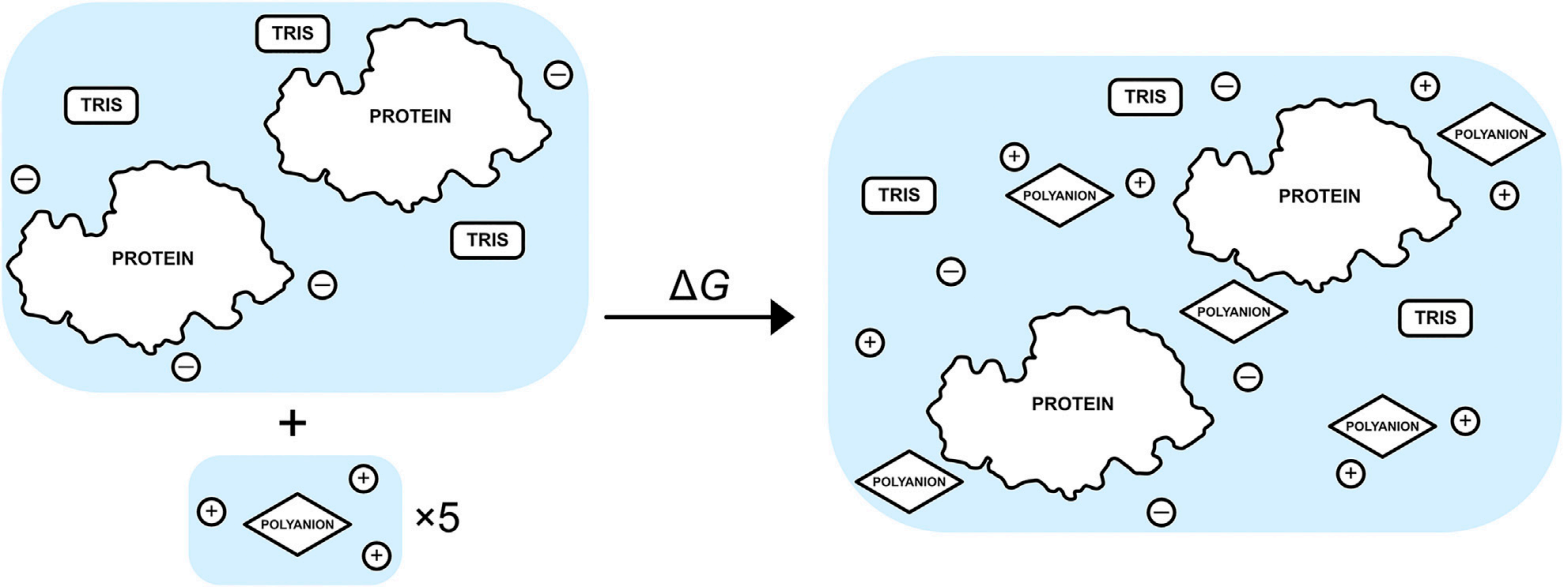
Schematic of the binding process between a set of five excipients, each with neutralising $Na^{+}$ ions, and a buffered protein-dimer system with neutralising $Cl^{-}$.

The free-energy change for binding five TPP or CIT excipient molecules to the buffered protein relative to an infinitely dilute excipient with their neutralising counterions is calculated using

$$\text{Δ}G=\sum_{X}\left[G_{\text{X},\text{bnd}}N_{\text{X}}+G_{\text{W},\text{bnd}}N_{{\text{W}}_{\text{X}},\text{bnd}}+G_{\text{W},\text{bulk}}\left(N_{{\text{W}}_{\text{X}},\text{dil}}-N_{{\text{W}}_{\text{X}},\text{bnd}}\right)\right]-\left[G_{\text{X},\text{dil}}N_{\text{X},\text{dil}}+G_{\text{W},\text{dil}}N_{{\text{W}}_{\text{X}},\text{dil}}\right]$$ (10)

where X is each type of solute, $N_{\text{X}}$ is the number of solute molecule X, and $N_{{\text{W}}_{\text{X}}}$ is the number of water molecules in the hydration shell of solute X. The subscripts “bnd”, “dil” and “bulk” refer to the bound or unbound dilute solutions and bulk water, respectively. The unbound protein dimer comprises chloride ions and five TRIS molecules (2-amino-2-hydroxymethyl-propane-1,3-diol) as shown in Figure 2. The five unbound excipients comprise a single dilute excipient molecule with three neutralising sodium counterions. The number of water molecules that are released from solvation shells go into bulk, and so they are assigned the free energy of pure water, which is obtained from a simulation of pure water.

### 2.5 Well-Tempered Metadynamics

Metadynamics allows for enhanced sampling compared to standard molecular dynamics (MD) simulations by forcing simulations to sample unexplored regions of free energy landscapes (Laio and Parrinello, 2002). Sampling is directed through the use of collective variables (CVs), whose choice depends on the system studied and the problem under consideration. Each CV is selected to most efficiently sample regions of interest, such as sampling dihedrals on a flexible peptide to find new and metastable conformations. To bias the system to previously unexplored regions, a biasing potential is used to add Gaussian functions to the potential, which depends on what regions of the potential energy landscape have been historically explored. This potential has the form

$$V\left(\vec{s},t\right)=\underset{k\tau <t}{\sum }W\left(k\tau \right)\text{exp}\left(-\overset{d}{\underset{i=1}{\sum }}\frac{{\left(s_{i}-s_{i}^{\left(0\right)}\left(k\tau \right)\right)}^{2}}{2\sigma_{i}^{2}}\right)$$ (11)

where $V\left(\vec{s,t}\right)$ is the history-dependent bias potential summed over all selected instantaneous collective variables $\vec{s}$ at time *t*. For each collective variable *i*, Gaussian functions (kernals) with width $\sigma_{i}$ and height W(kτ) are deposited every interval of *t* steps. Over a long enough time, the bias potential converges to minus the free energy $G\left(\vec{s}\right)$ (plus a constant *C*) as a function of all the CVs

$$V\left(\vec{s},t\to \infty \right)=-G\left(\vec{s}\right)+C$$ (12)

The CVs chosen are to efficiently fill metastable states and overcome barriers to neighbouring unexplored metastable states. To improve the convergence of metadynamics simulations, the height of the Gaussian is decreased with longer simulation time using well-tempered metadynamics (WT-MTD) (Barducci et al., 2008)

$$W\left(k\tau \right)=W_{0}\text{exp}\left(-\frac{V\left(s_{i}^{\left(0\right)}\left(k\tau \right),k\tau \right)}{k_{\text{B}}\text{Δ}T}\right)$$ (13)

where $W_{0}$ is the initial Gaussian height and ΔT is a temperature value selected to regulate the extent of exploration of free-energy, selected based on the bias factor γ as follows

$$\gamma =\frac{T+\text{Δ}T}{T}$$ (14)

This is the ratio between the temperature of the CVs and the system temperature. WT-MTD helps with free-energy barrier crossing by simulating CVs at higher temperatures and reduces noise by gradually reducing Gaussian heights.

#### 2.5.1 Reweighting Simulations From the Bias Potential

Because the bias potential in WT-MTD simulations is time-dependent, the weighting *w* for each simulation frame is calculated from the saved history-dependent bias potential applied at a given time frame *t* (Tiwary and Parrinello, 2015)

$$w\left(t\right)=\text{exp}\left(\frac{V\left(\text{u}\right)-V\left(s\vec{,}\;t\right)}{k_{\text{B}}T}\right)$$ (15)

where V(u) is the unbiased potential, equal to zero. Each statistical contribution to thermodynamics calculated here using MCC theory is weighted according to the above equation at a given simulation frame over all analysed frames using

$$A_{w}=\frac{A_{n}w_{n}+\sum_{t}^{n-1}A_{t}w_{t}}{w_{n}+\sum_{t}^{n-1}w_{t}}$$ (16)

Statistical values (*A*) used to calculate the free energy of excipients and water molecules at frame *t* are weighted ($A_{w}$) using a rolling average, where the weighted value of current frame *n* is added to the weighted values of all previous frames. The main influence of the biased potential between solutes and solvent is on the topographical entropy of water molecules, which is accounted for here. Protein values are not weighted because protein conformations are not sampled using a bias and the weightings would not greatly affect protein entropy, and in practice because the program used to calculate protein entropy does not account for biases.

### 2.6 Selection of Collective Variables and Metadynamics Variables

CVs are selected based on how efficiently the interactions between solutes can be sampled without having to sample more expensive molecular conformations. We therefore consider the number of contacts made between molecule types in the system. Three possible contact CVs are sampled: 1) protein-protein contacts of any side-chain oxygen or nitrogen between each protein, 2) polyanion-protein contacts of any oxygen on the polyanion with any oxygen or nitrogen on residue side chains and 3) any contacts between water oxygens and any O or N atom on a protein or polyanion. We therefore efficiently sample protein-protein interactions, protein-polyanion interactions and water-solute interactions. For each CV, the rational switching function, s(r) is used to set boundaries between a contact and no contact such that

$$s\left(r_{ij}\right)=\frac{1-{\left(\frac{r_{ij}-d_{0}}{r_{0}}\right)}^{6}}{1-{\left(\frac{r_{ij}-d_{0}}{r_{0}}\right)}^{12}}$$ (17)

where a contact $s\left(r_{ij}\right)$ between atoms *i* and *j* and distance $r_{ij}$ is 1 if less than distance $r_{0}$ and zero if beyond this distance. The switching function allows for the transition between 1 to 0 to be a continuous value for CV derivatives. A 10 Å neighbour list cutoff is used and updated every 2000 steps, with a contact being defined as two atoms between 0.5 and 6.5 Å distance. Contacts are described as O or N atoms within 6.5 Å with the switching function starting at 5 Å to gradually set the contact to zero at 6.5 Å. These contact definitions are used during metadynamics simulations. For post-processing of simulations, contacts are defined by coordination shells using the RAD algorithm as mentioned in Section 2.4.

The Gaussian width σ for each CV is selected based on the standard deviation observed from unbiased simulations. Contacts between solutes are set with σ=4 and for contacts with water molecules σ=30. The Gaussian height, W(kτ), is set to 1.5 $\text{kJ}\;{\text{mol}}^{-1}$ and Guassians are deposited every 500 steps. Simulations are run at T=298 K and the bias factor γ is set to 20. WT-MTD calculations for each system are performed across 25 simulations with differing starting poses of two lysozyme proteins and described in more detail next.

### 2.7 Multiple Walkers

**FIGURE 3:**
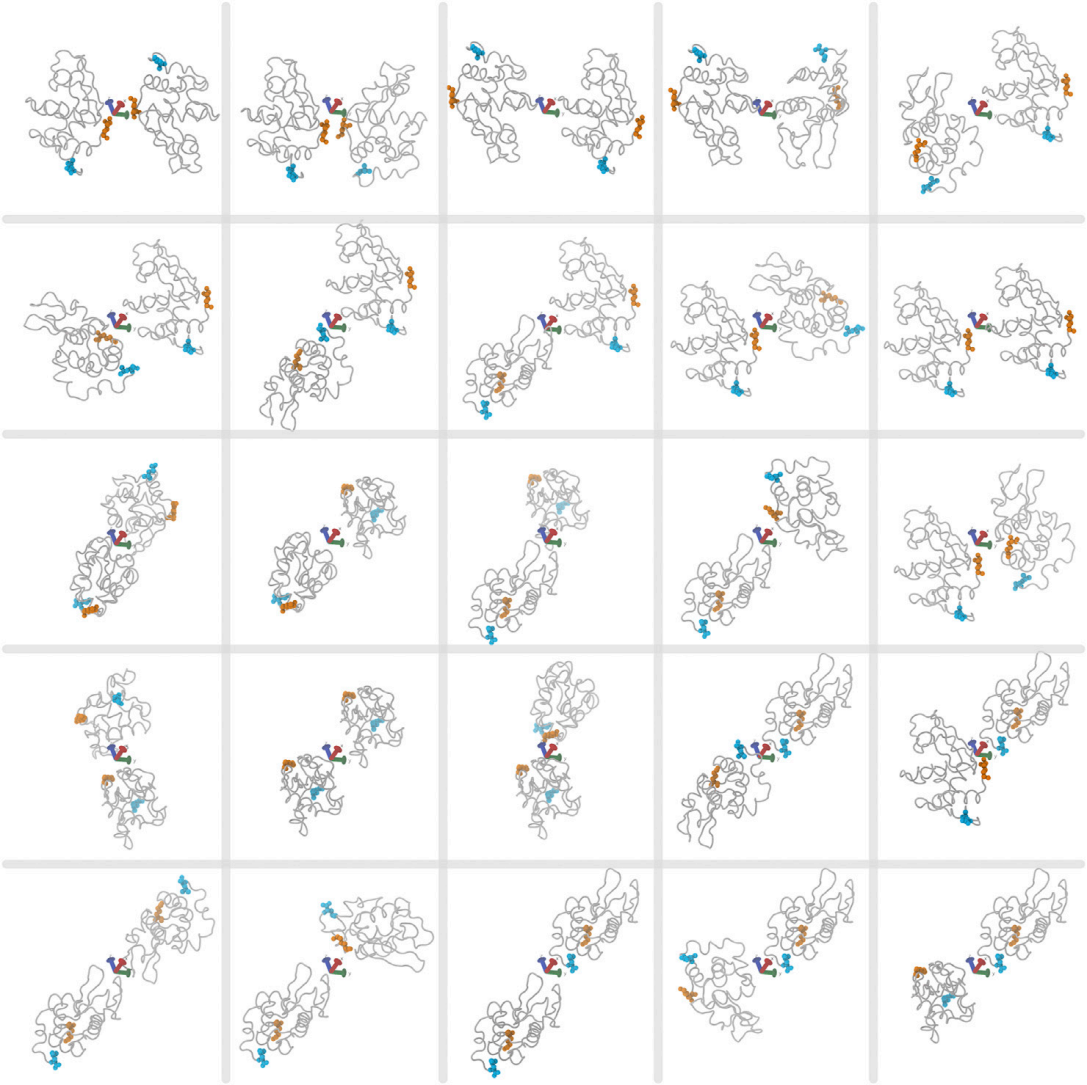
The 25 starting poses for two lysozyme proteins for each multiple-walker simulation. First and last protein residues in the sequence are highlighted in orange and cyan, respectively. The x,y and *z* axes (red, green, blue) lie at the origin of each simulation box.

Multiple walkers are multiple simulations running independently, but sharing information about already visited configurations along several CVs. Because we want to study non-specific interactions, which is characterised by many possibilities of weak or indirect interactions with the protein, there is no precise starting configuration to use. We thus consider multiple starting structures for sampling of interactions between proteins and polyanions. We use multiple walkers of 25 possible starting structures of various orientations between two proteins as shown in Figure 3. Starting poses are generated in VMD by sampling 90° orientations about the protein principal axes, giving 21 starting dimers with different protein surfaces facing each other. An additional four poses are included of the same protein surfaces facing each other but rotated about the dimer principal axes. While more starting poses and simulations would be required to achieve full protein-excipient sampling, this number of structures was chosen to resolve the different effects of the two excipients in a computationally efficient manner. A similar method to sample between multiple starting configurations is bias-exchange metadynamics, where many collective variables are sampled and exchanged between multiple simulations (Piana and Laio, 2007). Here we use multiple walkers instead of bias-exchange for a few reasons. Multiple walkers allow for simulations to start and run at different times, unlike with the bias-exchange method, where information is swapped every step between simulations. For multiple walkers, previously visited configurations over all steps and simulations are read in and biased toward unvisited configurations. Given that we use a high performance computing (HPC) cluster, our simulations can run independently without the need to wait for all 25 simulations to be simultanously running, therefore efficiently using the CPU resources. However, a disadvantage of multiple walkers is that only a few collective variables can be sampled. Guided by the previous experimental work (Bye and Curtis, 2019), we assume that sampling contacts between protein and excipients is sufficient to determine possible mechanisms for solute interactions. Lastly, because we use LAMMPS for per-atom energy, we are restricted to using multiple walkers rather than replica exchange, which is not currently supported for use between PLUMED and LAMMPS, as the latter uses temperature swaps, while PLUMED uses coordinate swaps between biased simulations.

### 2.8 System Setup

Structures of systems containing two lysozyme proteins surrounded by 14,400 water molecules to represent 100 mM protein concentration are created for three different conditions of excipient: tris(hydroxymethyl)aminomethane (TRIS) buffer only, citrate (CIT) with buffer and tripolyphosphate (TPP) with buffer. The two polyanion-containing systems have five polyanions to represent a concentration of approximately 20 mM and three TRIS molecules for approximately 10 mM buffer concentration for all systems. These conditions are comparable to experiment at the concentration where TPP precipitates lysozyme, while CIT does not (Bye and Curtis, 2019). Each system is created with 25 replicates of different starting poses of lysozymes with respect to each other. CIT or TPP are randomly assigned within a 30 Å radius sphere centered within a box of approximately 80 ${\text{Å}}^{3}$ for each system respectively. Each molecule in the system is neutralised with sodium or chloride ions depending on charge. Protein structure coordinates are taken from the Protein Data Bank with PDB-ID 2VB1 (Berman et al., 2000). The protein is ionised according to pKa of side chains at pH 9 using the PDB2PQR web server (Dolinsky et al., 2004). TPP and TRIS have a population of two charges at pH 9, so two TRIS molecules are set with 1+ charge and the other neutral, while for TPP one molecule is 4− charge and the other four have 5− charge. All five CIT molecules are 3− charge. Small-molecule geometries are built using Avogadro (Hanwell et al., 2012) and initial protein poses are created using VMD (Humphrey et al., 1996). Then all excipient, counterion and solvent molecules are populated around proteins, with at least an 8 Å layer of water molecules to the edge of the box using Packmol (Martínez et al., 2009). Additional systems of each excipient and counterion species surrounded by 900 water molecules are created to represent dilute reference systems with which to calculate changes in energy and entropy. Lysozyme is parametrised using ff14SB (Maier et al., 2015), water with TIP3P (Jorgensen et al., 1983) and and counter-ions with Joung and Cheetham parameters for TIP3P water (Joung and Cheatham, 2008). TRIS and CIT parameters are calculated using the AMBER GAFF (Wang et al., 2004) and TPP parameters with GAFF2 force field, partial charges are parametrised with the AM1-BCC charge method using antechamber (Wang et al., 2006), with additional parameters for the TPP O-P-O-P dihedral and P-O-P angle from Meagher et al. (Meagher et al., 2003).

### 2.9 Simulation Protocol

Each system is treated as a periodic box and initially minimized for 5,000 steepest descent steps with sander in AMBER18 (Pearlman et al., 1995). Topology and initial coordinates are converted to LAMMPS formatted files with InterMol (Shirts et al., 2017). A short 0.2 ns equilibration is conducted at NVT (number, volume, temperature) conditions to slowly heat the systems to 298 K and then equilibrated for 12 ns at constant NPT (number, pressure, temperature) conditions at 1 atm pressure with 1 fs time-steps in the LAMMPS simulation package (Plimpton, 1995). Restraints on protein Cα atoms are placed with a 500 $\text{kJ}\;{\text{mol}}^{-1}$ spring constant. After standard deviations for each CV are calculated, restraints on backbone atoms are removed and 10 ns of production simulations are performed. In total, 300 ns of equilibration and 250 ns production simulation time are conducted for each of the three systems over all 25 walkers. To maintain constant pressure and temperature, each system is controlled using a Nose-Hoover thermostat and barostat respectively. Temperature is relaxed every 0.2 ps and pressure is relaxed every 0.5 ps with an isotropic stress tensor across x,y,z box dimensions. For non-bonded iteractions, these are truncated to 8 Å, beyond which the particle-particle particle-mesh (PPPM) (Hockney and Eastwood, 1988) is used. All bonds to hydrogen atoms are constrained using the SHAKE algorithm (Ryckaert et al., 1977). Multiple walker well-tempered metadynamics sampling is performed using the PLUMED-2.0 plugin within LAMMPS (Tribello et al., 2014; Bonomi et al., 2019). Per-atom coordinates, forces, potential and kinetic energies are saved every 10 ps and a total of 20,000 frames are analysed for each system. Potential and kinetic energies are outputted per atom using the pe/atom and ke/atom flags respectively within LAMMPS. For potential energy, two and three-body energy terms are divided evenly over involved atoms and the long-range PPPM contributions are calculated using the method from Heyes (Heyes, 1994) within LAMMPS. For water and excipient free energy calculations, output files and topologies are read using the MDAnalysis python library (Michaud-Agrawal et al., 2011) and analysed with an in-house developed python program POSEIDON Beta V2 available at https://github.com/jkalayan/PoseidonBeta. For proteins, LAMMPS output files are first converted to CHARMM. psf and. dcd formats and stripped of solvent and excipients using CPPTRAJ (Roe and Cheatham, 2013) and protein entropy is calculated using code available at https://github.com/arghya90/CodeEntropy.

## 3 Results

### 3.1 Free Energy Change for the Formation of Each Lysozyme-Polyanion System

The total change in Gibbs energy, enthalpy and entropy for mixing each lysozyme-polyanion system from the individually separated polyanions and the protein-TRIS systems are shown in Table 1.

**TABLE 1:** Total ΔG, ΔH and TΔS and molecular components to form the lysozyme-polyanion systems.

	$\text{Δ}G\;/\;\text{kJ}\;{\text{mol}}^{-1}$		$\text{Δ}H\;/\;\text{kJ}\;{\text{mol}}^{-1}$		$T\text{Δ}S\;/\;\text{kJ}\;{\text{mol}}^{-1}$	
Species X	TPP	CIT	TPP	CIT	TPP	CIT
Lysozyme	−297	−226	−287	−267	10	−42
Polyanion	131	−76	136	−29	5	47
TRIS	−106	−85	−97	−80	9	6
$Na^{+}$	4	−4	4	−4	1	0
$Cl^{-}$	−3	−1	−3	−1	0	0
${\text{W}}_{\text{lysozyme}}$	4	−14	28	17	23	32
$W_{\text{polyanion}}$	−43	31	−84	30	−41	−1
${\text{W}}_{\text{TRIS}}$	34	40	38	43	4	3
$\text{W}\;{\text{Na}}^{+}$	2	−18	−28	−27	−29	−9
$\text{W}\;{\text{Cl}}^{-}$	−2	−1	0	1	2	2
Total	−276	−355	−293	−315	−17	39

**FIGURE 4:**
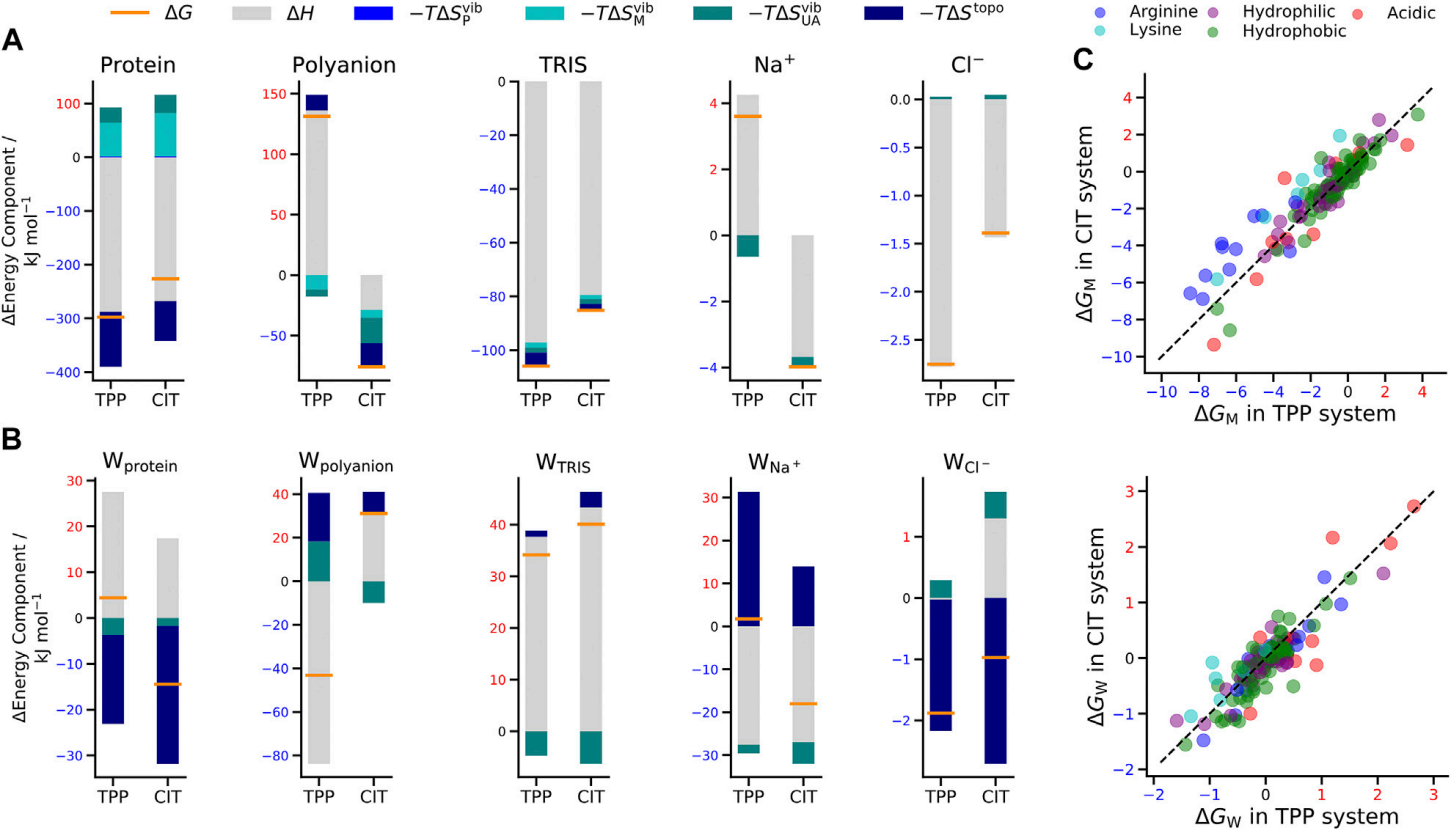
Change in free energy (orange line), enthalpy (grey bar) and entropy (bars in shades of blue) of **(A)** solutes and **(B)** water molecules around solutes in the TPP and CIT systems. **(C)** Change in free energy for CIT vs. TPP of each residue classified by type (top) and of water around each residue (bottom). The dashed line represents y=x.

Also included is a decomposition over all five kinds of solute in the system, namely protein lysozyme, polyanion TPP or CIT, buffer TRIS, counterions $Na^{+}$ or $Cl^{-}$ and all the water in the first hydration shell of each of species, denoted by W_*X*_. Estimation of standard errors in each thermodynamic property are extrapolated from a repeated simulation of dilute citrate as detailed and presented in Supplementary Tables S2 and S3. The dominant error is that of the protein ΔG at 12.1 $\text{kJ}\;{\text{mol}}^{-1}$ for both excipient systems, with slightly larger errors in ΔH and smaller errors in TΔS. Standard errors are also estimated based on the number of starting poses, which are analysed in five groups of five poses and are presented in Supplementary Table S4. These also show a similar largest contribution from the protein, with values of 15 $\text{kJ}\;{\text{mol}}^{-1}$ for TPP and 21 $\text{kJ}\;{\text{mol}}^{-1}$ for CIT systems. Further entropy decompositions are illustrated in Figures 4A,B according to the vibrational entropy at polymer, monomer and united-atom levels and the topographical entropy, which is conformational for proteins and polyanions and orientational for all molecules.

When interpreting the results, we refer to ΔG or ΔH values that are negative as stabilising the system, and conversely positive changes in components as destabilising the system. The opposite holds for TΔS. The total free energy change for polyanion mixing is large and negative, indicating that the CIT system is stabilised compared to TPP. The main contribution to the greater stability overall is lower CIT free energy compared to the destabilisation of TPP. This is offset by the reverse trend for hydrating water molecules around polyanions, with are stabilised near TPP but destabilised near CIT. Substantial energy-entropy compensation occurs in the stabilising of water molecules hydrating TPP, with water energy decreasing more than entropy. TRIS molecules are consistently stabilised for either polyanion, largely due to energy, while their surrounding waters are both destabilised. $Cl^{-}$ and $Na^{+}$ions are weakly affected upon mixing, although there is a greater change for the water molecules. The water solvating Na^+^ is stabilised in the CIT system because of enthalpy but destabilised in TPP due to enthalpy-entropy compensation because of a loss in orientational entropy.

Also of note is the number of contacts between each kind of species. Table 2 gives the number of contacts for the unbound systems, namely the separated excipient with $Na^{+}$ counterions and the two lysozymes buffered with TRIS and $Cl^{-}$ counterions, while Table 3 gives the contacts for the mixed polyanion-protein systems. A comparison of the tables shows the most noticeable change is a reduction in the number of contacts between $Na^{+}$ and polyanions upon binding for both polyanions, from 51.1 to 30.5 for TPP and from 16.3 to 7.7 for CIT. However, Table 1 indicates that this release of $Na^{+}$ into solution is only destabilising in the TPP system.

**TABLE 2:** Number of contacts between species in the separated excipient and lysozyme systems.

Species X	Lysozyme	TPP	CIT	TRIS	$Na^{+}$ (TPP)	$Na^{+}$ (CIT)	$Cl^{-}$
Lysozyme	3.8	-	-	5.7	-	-	0.9
TPP	-	-	-	-	51.1	-	-
CIT	-	-	-	-	-	16.3	-
TRIS	5.7	-	-	0.0	-	-	0.1
${\text{Na}}^{+}$	-	51.1	16.3	-	0.0	-	-
${\text{Cl}}^{-}$	0.9	-	-	0.1	-	-	-
Water	918.5	76.0	91.3	54.1	79.9	69.8	137.8

**TABLE 3:** Number of contacts between species in the polyanion-lysozyme systems.

	TPP system					
	Lysozyme	Polyanion	TRIS	**$Na^{+}$**	$Cl^{-}$	$N_{X}$
Lysozyme	15.1	17.5	7.3	3.2	0.8	2
Polyanion	17.5	0.1	8.1	30.5	0.0	5
TRIS	7.3	8.1	0.4	0.2	0.0	3
${\text{Na}}^{+}$	3.2	30.5	0.2	-	0.1	24
${\text{Cl}}^{-}$	0.8	0.0	0.0	0.1	0.0	18
Water	866.1	76.8	34.9	100.5	133.2	-

	CIT system					
Lysozyme	16.4	16.7	10.2	2.4	0.8	2
Polyanion	16.7	0.7	9.0	7.7	0.0	5
TRIS	10.2	9.0	0.6	0.1	0.0	3
$Na^{+}$	2.4	7.7	0.1	0.0	0.1	15
$Cl^{-}$	0.8	0.0	0.0	0.1	-	18
Water	864.1	77.1	31.1	77.3	133.0	-

**FIGURE 5:**
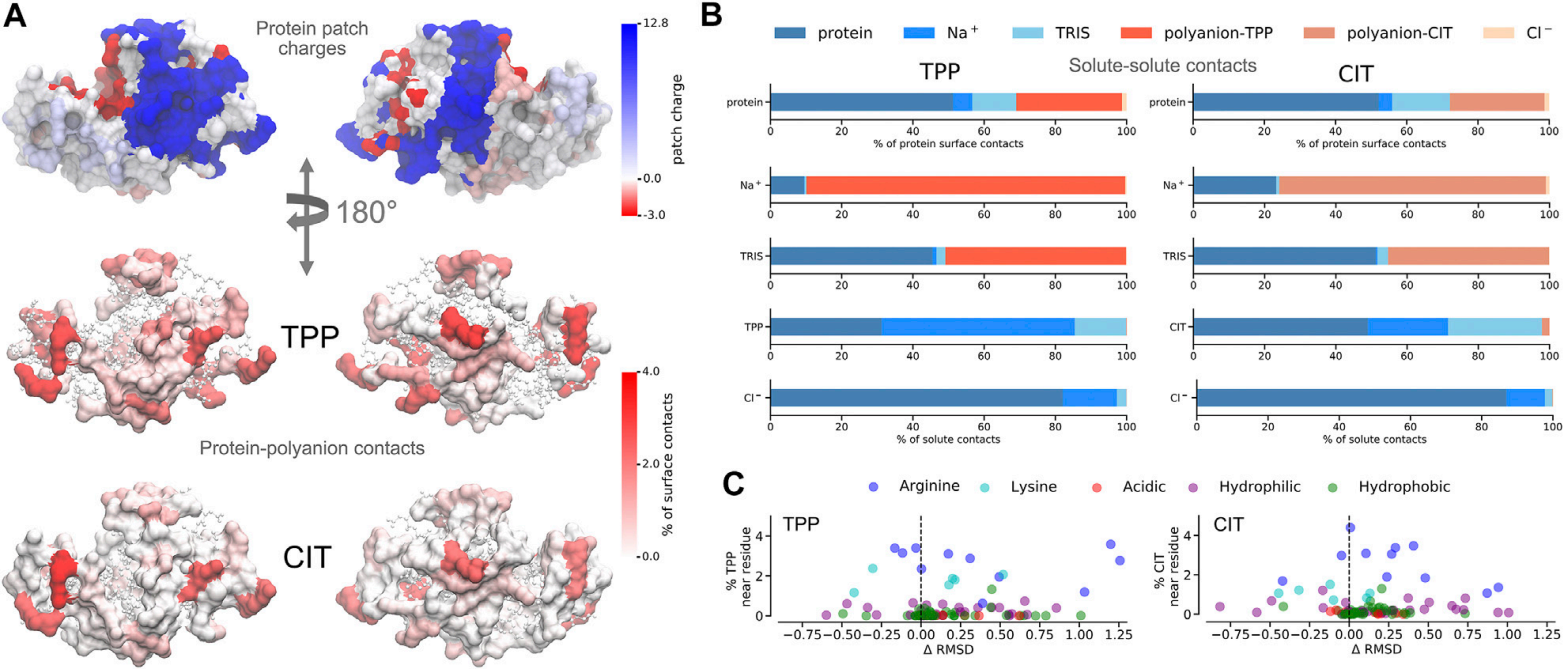
**A)** Charged patches on lysozyme at pH 9 for two opposing orientations (top). Average percentage of residue contacts (darker regions have more contacts) of polyanions mapped onto the protein surface for the same orientations (middle and bottom). Regions with no contacts are represented as CPK structures. **(B)** Percentages of solute-solute interactions in TPP (left) and CIT (right) systems. **C** Change of per-residue RMSD compared to the protein in buffer only plotted against percentages of polyanion interactions with each residue.

For proteins, there is a lower energy for both polyanions due to favorable electrostatic interactions between oppositely charges molecules, in particular, involving charged patches on lysozyme as shown in Figure 5. However, protein free energy is seen to be more stable with TPP than CIT. This is due to both energy and entropy (Table 1), with contributions from all components of protein entropy (Figures 4A,B). Interestingly, both protein conformational and surrounding water orientational entropy are larger in the presence of polyanions, which suggest weaker interactions of the protein with its environment. Correspondingly, an increase in residue RMSD is observed in the CIT and TPP systems in Figure 5C. Water around proteins is destabilised in both energy and entropy in the presence of polyanions, bringing about slightly more stabilisation for CIT than TPP. Further decomposition of the change in free energies for each kind of residue according to charge, hydrophilicity and hydrophobicity and for their surrounding waters are plotted in Figure 4C. The free energy of basic residues, particularly Arg, is reduced for both polyanions but more so in the TPP system than CIT system. The free energy of acidic residues is generally more destabilised with TPP than with CIT.

### 3.2 Interactions Between Solutes

Given the free-energy destabilisation for TPP but stabilisation for CIT, we assess further how interactions with other solute molecules may cause these differences in free energy. In dilute solution TPP has more UA contacts with $Na^{+}$, namely 10.2 contacts per TPP with an average TPP charge of −4.8, suggesting strongly bound $Na^{+}$ as calculated from the number of contacts between molecules in Tables 2, 3. Dilute CIT has 3.3 contacts with $Na^{+}$, matching its −3 cha^+^rge and suggesting $Na^{+}$ is not as tightly bound. In protein solutions, TPP and CIT have approximately 60 and 50% reductions in contacts with $Na^{+}$ respectively, but only TPP is destabilised.

From Figure 5A, both polyanions have similar numbers of contacts in similar regions of positively charged patches on the protein surface. Patches are generated on the protein using Poisson-Boltzmann electrostatics as described in previous work (Kalayan et al., 2020). CIT has overall weaker interactions over the protein surface, while TPP interacts with fewer regions of the protein but with higher occupancy. From Figure 5B, TPP still has a high percentage of $Na^{+}$ contacts compared with all other solutes, even though TPP forms more contacts with the protein that CIT. Similarly, $Na^{+}$ interacts more with TPP than the protein or CIT. As TPP has more strongly bound $Na^{+}$, we assume that the loss of some bound counterions destabilises TPP. Interactions between TPP and basic residues stabilise the protein, but do not appear to stabilise TPP. For CIT, the loss in half of the interacting $Na^{+}$ is compensated by the interactions with basic residues which stabilise CIT.

As to why TPP remains bound to the lysozyme surface even though it is destabilised, we observe stabilisation of residues that TPP interacts with most frequently from Figures 5C, 4C, where highest percentage occupied residues are also reduced the most in free energy. Therefore protein stabilisation may prevent TPP from being released into solution. Another possible reason for TPP remaining in the bound state may be attributed to surrounding water molecules as described in more detail next.

### 3.3 Specific Residue Interactions of Water Molecules

**FIGURE 6:**
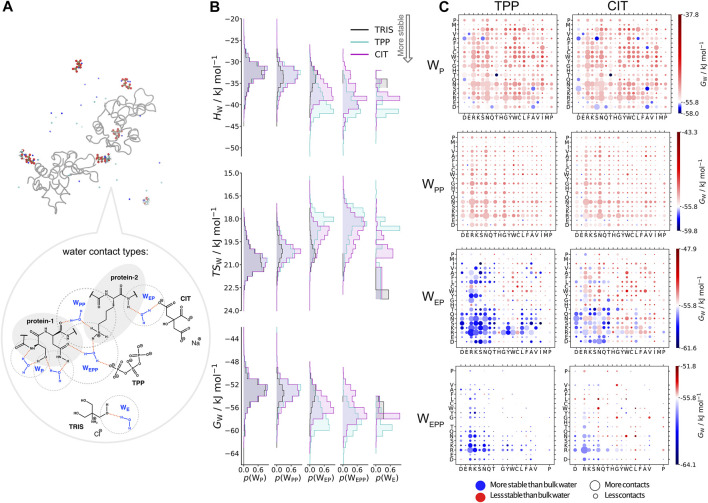
**A)** Water types ($W_{\text{P}}$, $W_{\text{PP}}$, $W_{\text{EP}}$, $W_{\text{EPP}}$, $W_{\text{E}}$) based on what solute types are in their coordination shell (grey dashed circles). **(B)** Distribution of water types energy, entropy and free energy (top to bottom) for each system: TRIS only buffer (black), buffer + TPP (cyan) and buffer + CIT (magenta). **(C)** Water free energy for each water type in TPP and CIT systems is based on which residue pairs are in a water coordination shell.

In Figure 6, water molecules are analysed based on the solutes in their first coordination shell. Total numbers of each water type are given in Table 4. By studying interactions of water with solutes, we can also infer which solutes are in close proximity to one another from the reduction in water coordination. Representation of each water type is shown in part A. In part B, distributions of water energy and entropy for each water type are shown, where water molecules interacting with just protein residues ($W_{\text{P}}$ and $W_{\text{PP}}$) have similar distributions for all three systems. However, when a polyanion is in the system, $W_{\text{EP}}$ and $W_{\text{EPP}}$ water molecules are most energetically stabilised in the TPP system. Some water molecules are stabilised in the CIT system, but to a greater extent many water molecules remain less stable compared to bulk water.

**TABLE 4:** Number of water-molecule environments for each protein-excipient system.

Water contact type	TRIS	TPP	CIT
${\text{W}}_{\text{P}}$	906.9	843.1	837.2
${\text{W}}_{\text{PP}}$	5.2	15.0	15.4
${\text{W}}_{\text{EP}}$	6.4	7.8	11.0
${\text{W}}_{\text{EPP}}$	0.0	0.3	0.5
Total	918.5	866.1	864.1

Part C in Figure 6 shows the water environments based on their interactions with the nearest two residues, ordered by acidic, basic, uncharged polar and non-polar from left to right. More destabilised $W_{\text{EP}}$ and $W_{\text{EPP}}$ are present for CIT compared to TPP because excipients are near more hydrophobic residues than in the TPP system. $W_{\text{P}}$ and W$W_{\text{PP}}$ water molecules generally are less stable than in bulk. The distribution of neighbouring residues interacting with W$W_{\text{P}}$ water molecules is less specific than residues involved in ${\text{W}}_{\text{PP}}$ interactions. This also indicates that PPIs between lysozyme molecules without excipients involved occur mostly between hydrophilic residues. In the presence of polyanions, protein-protein interactions occur mainly between arginine residues. However, in the presence of CIT, PPIs also occur between hydrophobic residues, which explains the distribution of less stable $W_{\text{EPP}}$ water molecules in the CIT system in part B.

## 4 Discussion

Protein precipitation is often characterised by the propensity for ions to “salt-out” a protein. Furthermore, the ion-specificity to cause precipitation can be ranked in the Hofmeister series (Hofmeister, 1888). The mechanism by which protein precipitation occurs is generally considered to be caused by ions forming stronger interactions than proteins with surrounding solvent. It is assumed that stronger ion-solvent interactions leads to a more structured water HB network, explaining the designation of such ions as kosmotropes (Collins, 1997). It is the loss of water at the protein surface that then drives PPIs between hydrophobic regions. Suggested mechanisms for protein precipitation assume that ions do not interact preferentially with the protein surface, and instead remain fully hydrated in solution due to strong interactions with water. In the case of multivalent anions or cations, however, these ions bind onto the protein surface as shown by the change in net protein charge in experimental measurements of their zeta potentials (Matsarskaia et al., 2016; Roosen-Runge et al., 2013; Matsarskaia et al., 2018). At the concentration when the net charge of a globular protein is neutral, the propensity to precipitate is high because repulsive interactions are screened by bound ions. This phenomenon is observed for polyvalent cations ($Y^{3+}$, $La^{3+}$, $Al^{3+}$, $Fe^{3+}$) when interacting with net-negatively charged globular proteins (Roosen-Runge et al., 2013). Yet similar studies with polyvalent anions shows selectivity between anions to salt out proteins, even though ion binding to the protein and net-neutrality both occur universally (Bye and Curtis, 2019).

To understand why ion specific protein precipitation occurs, the change in both the stability and contacts between solutes molecules is studied for conditions where TPP causes lysozyme to precipitate but CIT does not. Stability is assessed by the change in Gibbs free energy for mixing lysozyme with each excipient and the contributions of all molecules involved. This analysis shows several differences between TPP and CIT-containing systems. First we observe TPP destabilisation upon interaction with lysozyme, caused by the release of half of the strongly bound $Na^{+}$ ions surrounding TPP. Although unfavourable for TPP, protein binding occurs due to the overall stabilisation of the protein, which is stronger than TPP destabilisation. Conversely, CIT is not destabilised upon protein binding, even with the loss of $Na^{+}$ ions, which are not strongly bound to CIT. However, cross-linking with another protein does not occur with CIT because this is not favourable for the surrounding water molecules.

The stability and contacts of water surrounding each solute reveals the stabilisation of water around TPP, but overall destabilisation around CIT. Upon binding to the protein, TPP does not lose any water-molecule contacts. Instead, water-molecule energy is stabilised but entropy is lost, therefore forming a more structured HB network around TPP and agreeing with the description of TPP as a kosmotropic ion. Thus instead of water being stabilised upon release into solution as is usually the case for PPIs, water is stabilised by forming strong interactions with protein-bound TPP when replacing released $Na^{+}$ counterions. Water entropy around CIT does not change greatly (TΔS=−1 kJ mol^-1^) when CIT interacts with the protein but the contacts of CIT with water moleculesreduce by ∼15%. The lack of strongly bound, ordered water molecules suggests that CIT behaves as a chaotropic salt, thereby not salting out the protein. Overall, the stabilisation of water around lysozyme upon CIT binding maintains lysozyme solubility.

Although water is slightly destabilised around lysozyme in the presence of TPP, it does not seem to be destabilised enough to cause precipitation via hydrophobic interactions. Instead the combination of overall solvent and protein stabilisation upon TPP binding is expected to drive binding via cross-linking with other proteins at moderate TPP concentration. In the conditions simulated here, five polyanion molecules per lysozyme pair closely represents a 20 mM concentration. There are a total of 34 basic residues on the lysozyme pair at pH 9 with a net charge of 16+, therefore giving many vacancies for TPP cross-linking. At 100 mM TPP concentration, experiment shows that lysozyme is fully resolublised into solution (Bye and Curtis, 2019) and there are approximately 29 TPP molecules per lysozyme pair. Each basic residue can be bound individually at 100 mM TPP, and therefore no cross-linking between proteins would be required. To more conclusively explain the experimental data, much longer simulations with more starting poses would be required that can explicitly account for direct lysozyme-lysozyme binding and cross-linking of lysozyme molecules by excipients at varying concentrations of each excipient. Adequately addressing such questions may require coarse-grain simulations to achieve the necessary sampling. At the same time, greater model accuracy may also be required by accounting for the possibility of variable protonation states of amino acids and excipients arising from their changing environments using constant pH simulations. This makes clear that much work remains in order to fully understand the effects of excipients on protein behaviour.

## 5 Conclusion

Lysozyme-excipient systems have been studied to understand how excipients differentially interact with lysozyme using a statistical-mechanics based free-energy method called Energy-Entropy Multiscale Cell Correlation that calculates the energy and entropy of each solute and hydration shell water molecules. Simulations are conducted using multiple walker metadynamics simulations followed by reweighting to give a Boltzmann distribution. We observe different contacts and thermodynamic properties when the excipient TPP interacts with lysozyme compared to the excipient CIT. A possible mechanism by which TPP precipitates lysozyme is suggested to be due to the stabilisation in free energy of both lysozyme and solvent surrounding TPP upon TPP interacting with basic residues and the release of bound $Na^{+}$ ions.

## Data Availability

The raw data supporting the conclusions of this article will be made available by the authors, without undue reservation.
