# Supplementary material for: Thermodynamic Origin of Differential Excipient-Lysozyme Interactions
Source: Front Mol Biosci. 2021 Jun 11;8:689400. doi: 10.3389/fmolb.2021.689400 (PMC8226134; doi:10.3389/fmolb.2021.689400)
Supplement: Supplementary file 1 [file DataSheet1.PDF]

Supplementary Material

Table S1. Axes and origin of axes (where COM is centre-of-mass) to rotate forces about for each species and length scale when calculating translational (top) and rotational (bottom) entropy.

Translations

Length scale	Species	Axes	Origin of Axes
Polymer	Protein	Principal	COM
Monomer	Residue	Polymer Principal	Polymer COM
	Excipient	Principal	COM
United atom	UA + bonded Hs	Average vector of bonded Hs and two arbitrary orthogonal vectors	Heavy atom
	UA with no bonded Hs	Arbitrary	Arbitrary

Rotations

Length scale	Species	Axes	Origin of Axes
Polymer	Protein	Principal	COM
Monomer	Residue	Principal	Average of three backbone bonded atoms
	Excipient	Principal	COM
United atom	UA + bonded Hs	Average vector of bonded Hs and two arbitrary orthogonal vectors	Heavy atom
	UA with no bonded Hs	N/A	N/A

ERROR ESTIMATION

Standard errors (SE) in energy and entropy for the solute and surrounding water molecules are estimated from the standard deviation (σ) in thermodynamic properties in a dilute citrate solution

$$\sigma = \sqrt{\frac{\sum_{i=1}^{n}(x_i - \mu)^2}{n}} \tag{S1}$$

$$\mathrm{SE} = \frac{\sigma}{\sqrt{n}} \tag{S2}$$

where μ is the mean and $n = 4$ is the number of simulations. For each repeat, 5000 frames from 10 ns simulations are analysed, errors are given in Table S2. To extrapolate errors to lysozyme, tripolyphosphate, and surrounding water molecules, the SE is multiplied by the square root of the number of united-atoms of a species in the system divided by the number in single citrate. For chloride ions its surrounding solvent, the errors are taken as the same as for sodium ions. Estimated standard errors are given in Table S3.

Standard errors are also estimated for the Gibbs free energy of protein-excipient mixing by dividing up the 25 poses into five groups of five and calculating the standard error from these using Equations S1 and

S2. The standard errors obtained are reported in Table S3 and indicate the dependence of ΔG values on the number of poses used.

Table S2. Mean (μ) and Standard Error (SE) of G, H and TS for a Set of Four Simulations of Citrate and Sodium in Water

		G / kJ mol^{-1}		H / kJ mol^{-1}		TS / kJ mol^{-1}	
Species	N_X	μ	SE	μ	SE	μ	SE
CIT	1	-1033.2	1.0	-940.0	1.3	93.2	0.5
Na^+	3	-1221.2	0.6	-1183.7	0.6	37.5	0.1
$W_{citrate}$	18.0	-820.9	0.3	-522.2	0.6	391.8	0.4
W_{Na^+}	13.6	-809.5	0.2	-510.9	0.2	298.6	0.1

Table S3. Standard Error (SE) of G, H and TS for Each Set of Solute and Their Surrounding Water Molecules in Each Lysozyme-Polyanion Mixture

	SE for G / kJ mol^{-1}		SE for H / kJ mol^{-1}		SE for TS / kJ mol^{-1}	
Species X	TPP	CIT	TPP	CIT	TPP	CIT
lysozyme	12.1	12.1	15.6	15.6	5.7	5.7
polyanion	2.2	2.2	2.8	2.8	1.0	1.0
TRIS	1.3	1.3	1.7	1.7	0.6	0.6
Na^+	1.7	1.4	1.8	1.4	0.1	0.1
Cl^-	1.5	1.5	1.6	1.6	0.1	0.1
$W_{lysozyme}$	2.4	2.4	4.3	4.3	2.8	2.8
$W_{polyanion}$	3.0	3.0	5.4	5.4	3.5	3.5
W_{TRIS}	0.5	0.5	0.9	0.8	0.6	0.5
W_{Na^+}	0.6	0.5	0.5	0.4	0.3	0.3
W_{Cl^-}	0.6	0.6	0.5	0.6	0.3	0.3

Table S4. ΔG Components of Protein-Excipient Mixing and Standard Errors (SE) for the Five Groups of Five Starting Poses

	ΔG / kJ mol^{-1}											
	poses 1 to 5		poses 6 to 10		poses 11 to 15		poses 16 to 20		poses 21 to 25		SE	
Species X	TPP	CIT	TPP	CIT	TPP	CIT	TPP	CIT	TPP	CIT	TPP	CIT
lysozyme	-335	-237	-232	-217	-277	-287	-284	-250	-311	-349	15	21
polyanion	118	-73	144	-76	158	-67	158	-71	129	-57	7	3
TRIS	-92	-73	-83	-96	-119	-93	-106	-131	-132	-54	8	12
Na^+	33	-2	19	-11	14	-12	30	-22	9	-12	4	3
Cl^-	-3	-1	0	1	-4	-3	-3	-1	-3	-3	1	1
$W_{lysozyme}$	13	-2	-12	15	-32	-39	-45	-19	-20	-42	9	10
$W_{polyanion}$	-9	54	-44	64	-34	70	-10	70	-10	46	7	4
W_{TRIS}	25	28	19	32	24	29	24	34	26	24	1	2
W_{Na^+}	7	-14	2	-8	2	-9	8	-4	8	-6	1	2
W_{Cl^-}	-13	-9	-12	-12	-16	-12	-14	-12	-17	-10	1	1
